# Advances of Targeted Therapy for Hepatocellular Carcinoma

**DOI:** 10.3389/fonc.2021.719896

**Published:** 2021-07-26

**Authors:** Mengke Niu, Ming Yi, Ning Li, Kongju Wu, Kongming Wu

**Affiliations:** ^1^ Department of Oncology, Tongji Hospital of Tongji Medical College, Huazhong University of Science and Technology, Wuhan, China; ^2^ Department of Medical Oncology, The Affiliated Cancer Hospital of Zhengzhou University, Henan Cancer Hospital, Zhengzhou, China; ^3^ Department of Nursing, Medical School of Pingdingshan University, Pingdingshan, China

**Keywords:** hepatocellular carcinoma, targeted therapy, tyrosine kinase inhibitors, immune checkpoint inhibitors, clinical trials

## Abstract

Hepatocellular carcinoma (HCC) is one of the common and fatal malignancies, which is a significant global health problem. The clinical applicability of traditional surgery and other locoregional therapies is limited, and these therapeutic strategies are far from satisfactory in improving the outcomes of advanced HCC. In the past decade, targeted therapy had made a ground-breaking progress in advanced HCC. Those targeted therapies exert antitumor effects through specific signals, including anti-angiogenesis or cell cycle progression. As a standard systemic therapy option, it tremendously improves the survival of this devastating disease. Moreover, the combination of targeted therapy with immune checkpoint inhibitor (ICI) has demonstrated more potent anticancer effects and becomes the hot topic in clinical studies. The combining medications bring about a paradigm shift in the treatment of advanced HCC. In this review, we presented all approved targeted agents for advanced HCC with an emphasis on their clinical efficacy, summarized the advances of multi-target drugs in research for HCC and potential therapeutic targets for drug development. We also discussed the exciting results of the combination between targeted therapy and ICI.

## Introduction

Hepatocellular carcinoma (HCC) accounts for approximately 75%-85% of all primary liver cancer (1). Several risk factors such as chronic hepatitis B virus (HBV) and hepatitis C virus (HCV) infections, autoimmune hepatitis, alcohol abuse, diabetes, obesity induce liver injury and produce an inflammatory environment, which lead to hepatocyte necrosis, repeated regeneration and chromosomal instability (2, 3). The gradual accumulation of genetic and epigenetic abnormalities in this background plays an essential role in hepatocarcinogenesis (4). As curative treatments, surgical resection, radiofrequency ablation (RFA), transarterial chemoembolization (TACE) and liver transplant (LT) prolong the survival of HCC patients at early-or intermediate-stages (5–7). However, the high incidence of recurrence indicates poor survival prospects (8–11). Besides, most of HCCs are diagnosed at an advanced stage due to its insidious onset and rapid progression (7). Palliative treatments are therefore crucial in the management of advanced HCC. The efficacy of systemic chemotherapy for advanced HCC is disappointing (12).

**Figure 1:**
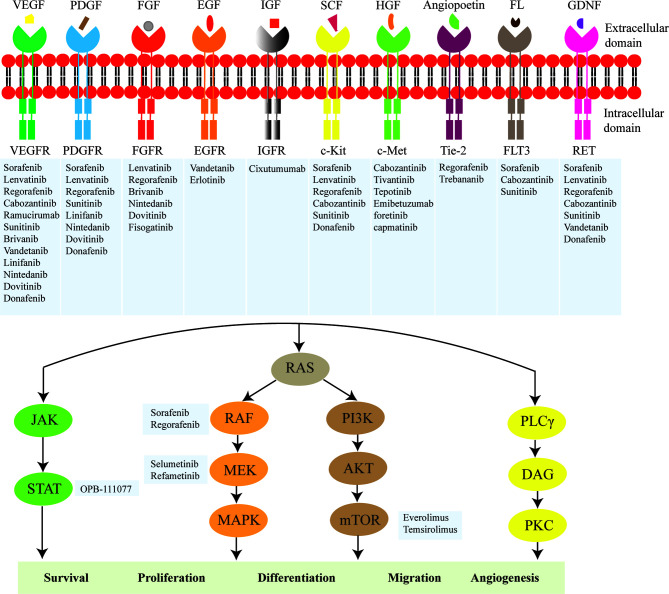
Main molecules of targeted therapy for hepatocellular carcinoma (HCC). The major targets include vascular endothelial growth factor receptor (VEGFR), platelet-derived growth factor receptor (PDGFR), FGF receptor (FGFR), epidermal growth factor receptor (EGFR), insulin-like growth factor receptor (IGFR), c-Kit, hepatocyte growth factor receptor (c-Met), Tie-2, FLT3, RET, RAF, MEK, STAT, and mTOR. The key mechanisms are to inhibit the activity of tyrosine kinase in the intracellular domain of the receptor tyrosine kinase or directly block the transduction of downstream signals involved in cell survival, proliferation, differentiation, migration and angiogenesis.

In recent years, molecular biology techniques are rapidly developing, such as whole exome sequencing, copy number analyses, mRNA-seq, miRNA-seq, methylomics and proteomics (13–15). Multiplex molecular profiling of HCC deepens on the understanding of aberrant molecular events and pivotal signaling pathways associated with the development of HCC, especially tyrosine kinase-related signaling (14). In general, tyrosine kinases can be classified as receptor tyrosine kinases (RTKs) and non-receptor tyrosine kinases (nRTKs) (16). RTKs transmit extracellular signals and nRTKs mediate intracellular communications (16). RTKs are receptors of a variety of subfamilies, including vascular endothelial growth factor receptor (VEGFR), platelet-derived growth factor receptor (PDGFR), epidermal growth factor receptor (EGFR), fibroblast growth factor receptor (FGFR), hepatocyte growth factor receptor (HGFR), Tie-2 and RET (**Figure 1**) (17–20). RTK consists of an extracellular domain that binds specific ligand, a transmembrane domain and an intracellular domain with tyrosine kinase activity (21). The binding of RTK to its ligand phosphorylates tyrosine residues of target protein and regulates a series of biochemical processes through corresponding downstream signaling pathways (17, 18). Functional mutations, genomic amplification, chromosomal rearrangements and/or autocrine activation lead to oncogenic activation of RTK, ultimately leading to carcinogenesis, invasion, metastasis, and angiogenesis (17, 22, 23). The emergence of tyrosine kinase inhibitors (TKIs) has become a promising targeted therapeutic strategy (24, 25). TKIs can enter cells and interact with the intracellular domain of multiple receptors and other intracellular signaling molecules, blocking the phosphorylation of tyrosine residues and the activation of various downstream signaling pathways such as the Ras/Raf/MEK/MAPK and PI3K/AKT/mTOR (16).

Given the current investigation, multiple drugs have been approved for advanced HCC (**Table 1**). The emergence of targeted therapy has transformed the therapeutic landscape of advanced HCC (5, 24, 26–28). Despite advances in targeted therapy, overall response rate and 5-year survival rate remain unsatisfactory (29). The inevitable development of drug resistance and toxicity, and the absence of specific biomarkers to screen patients sensitive to these agents, have spurred the further exploration of novel therapeutic targets and strategies (29–31).

**Table 1:** Principal clinical trials for the FDA-approval of targeted and immunotherapeutic drugs for HCC.

Drugs	Main targets	Treatment line	Pivotal study	Study design	Results	Approval time
Sorafenib	VEGFRs, PDGFR-β, c-Kit, FLT3, RET	First-line	NCT00105443	Phase III, sorafenib *vs*. placebo	OS: 10.7 *vs*. 7.9 months (HR 0.69; 95%CI: 0.55-0.87, *p*<0.001)	2007
					Time to radiologic progression: 5.5 *vs*. 2.8 months (HR 0.58; 95%CI: 0.45-0.74, *p*<0.001)	
					ORR:2% *vs*. 1%	
Lenvatinib	VEGFR1-3, FGFR1-4, PDGFR-α, RET, c-Kit	First-line	NCT01761266	Phase III, lenvatinib *vs*. sorafenib	OS: 13.6 *vs*. 12.3 months (HR 0.92; 95%CI: 0.79-1.06)	2018
					PFS: 7.4 *vs*. 3.7 months (HR 0.66; 95%CI: 0.57-0.77, *p*<0.0001)	
					TTP: 8.9 *vs*. 3.7 months (HR 0.63; 95%CI: 0.53-0.73, *p*<0.0001)	
					ORR: 40.6% *vs*. 12.4%	
Atezolizumab plus Bevacizumab	PD-L1 VEGF	First-line	NCT03434379	Phase Ib, atezolizumab plus bevacizumab *vs*. sorafenib	Survival rates at 12 months: 67.2% *vs*. 54.6%	2020
					PFS: 6.8 *vs*. 4.3 months (HR 0.59; 95%CI: 0.47-0.76, *p*<0.001)	
					ORR: 33.2% *vs*. 13.3%	
Regorafenib	VEGFR1-3, PDGFR-β, FGFR1, Tie-2, c-Kit, RET, B-RAF	Second-line	NCT01774344	Phase III, regorafenib *vs*. placebo	OS: 10.6 *vs*. 7.8 months (HR 0.63; 95%CI: 0.50-0.79, *p*<0.0001)	2017
					PFS: 3.1 *vs*. 1.5 months (HR 0.46; 95%CI: 0.37-0.56, *p*<0.0001)	
					ORR: 11% *vs*. 4%	
Cabozantinib	VEGFR2, c-Met, RET, c-Kit, AXL, FLT3	Second-line	NCT01908426	Phase III, cabozantinib *vs*. placebo	OS: 10.2 *vs*. 8.0 months (HR 0.76; 95%CI: 0.63-0.92, *p*=0.005)	2019
					PFS: 5.2 *vs*. 1.9 months (HR 0.44; 95%CI: 0.36-0.52, *p*<0.001)	
					ORR: 4% *vs*. <1%	
Ramucirumab	VEGFR2	Second-line	NCT02435433	Phase III, ramucirumab *vs*. placebo	OS: 8.5 *vs*. 7.3 months (HR 0.71; 95%CI: 0.531-0.949, *p*=0.0199)	2019
					PFS: 2.8 *vs*. 1.6 months (HR 0.452; 95%CI: 0.339-0.603, *p*<0.0001)	
					ORR: 5% *vs*. 1%	
Nivolumab	PD-1	Second-line	NCT01658878	Phase I/II, nivolumab	ORR: The dose-expansion phase 20% (95% CI: 15-26)	2017
					The dose-escalation phase 15% (95% CI: 6-28)	
Pembrolizumab	PD-1	Second-line	NCT02702414	Phase II, pembrolizumab	ORR: 17%	2018
					1 (1%) complete and 17 (16%) partial responses	
Nivolumab plus Ipilimumab	PD-1 CTLA-4	Second-line	NCT01658878	Phase I/II, Nivolumab Ipilimumab	ORR: arm A: 32% arm B: 27% arm C: 29% OS: arm A: 22.8 months arm B: 12.5 months arm C: 12.7 months	2020

HCC, hepatocellular carcinoma; VEGFR, vascular endothelial growth factor receptor; PDGFR, platelet-derived growth factor receptor; c-Kit, stem cell factor receptor; FLT3, FMS-like tyrosine kinase-3; RET, rearranged during transfection; OS, overall survival; ORR, objective response rate; FGFR, fibroblast growth factor receptor; PFS, progression-free survival; TTP, time to progression; PD-L1, programmed cell death ligand 1; VEGF, vascular endothelial growth factor; Tie-2, tyrosine kinase with immunoglobulin-like and epidermal growth factor homology domains; c-Met, hepatocyte growth factor receptor; PD-1, programmed cell death protein 1; CTLA-4, cytotoxic T-lymphocyte-associated antigen 4.

Effective combination therapy is needed due to the limited efficacy of monotherapy. Recent studies have shown that combinations of multiple therapeutic regimens demonstrated superior efficacy to monotherapy, particularly combination of targeted therapy with immune checkpoint inhibitor (ICI) (32). Notably, the approval of atezolizumab plus bevacizumab as the first-line setting for patients with unresectable or metastatic HCC alters the outlook for this disease. This review focused on the advances of targeted therapy for advanced HCC.

## Approved Targeted Therapeutic Agents for HCC

### First-Line Setting

#### Sorafenib

Sorafenib is an oral multi-targeted TKI, which exerts dual antitumor effects (33). This drug not only directly suppresses tumor cells proliferation by blocking RAF/MEK/ERK and JAK/STAT signaling pathways, but also inhibits tumor angiogenesis by targeting VEGFRs, PDGFR-β, c-Kit, FLT3, RET (33, 34). In the Sorafenib HCC Assessment Randomized Protocol (SHARP) trial, in comparison to placebo arm, sorafenib arm showed prolonged overall survival (OS) (10.7 months *vs* 7.9 months; HR 0.69; p<0.001) and time to radiologic progression (5.5 months *vs* 2.8 months; HR 0.58; p<0.001) (35). Based on the results, sorafenib was approved by FDA for the first-line treatment of advanced HCC in 2007. The similarly promising results were displayed in another phase III Oriental trial. The study also showed a significant improvement in median OS (6.5 months *vs* 4.2 months; HR 0.68; p=0.014) and time to progression (TTP) (2.8 months *vs* 1.4 months; HR 0.57; p=0.0005) in patients treated with sorafenib compared with placebo (36). Unfortunately, the treatment-related adverse events led to dose reductions in small fraction of patients and rarely needed interruptions (36).

#### Lenvatinib

Lenvatinib is an oral multi-kinase inhibitor targeting VEGFR1-3, FGFR1-4, PDGFR-α, RET and c-Kit (37). Lenvatinib was approved by the FDA in 2018 as first-line treatment for advanced HCC. The approval is based on an open-label, phase III, multicenter, non-inferiority trial (38). The previous phase II clinical trial had shown positive results of lenvatinib for the treatment of HCC (39). Then, the further phase III, non-inferiority trial was performed to compare the efficacy and safety of lenvatinib *versus* sorafenib in HCC patients (38). As first-line treatment, lenvatinib was non-inferior to sorafenib in OS (13.6 months *vs* 12.3 months; HR 0.92) (38). Furthermore, lenvatinib showed a significant improvement in progression-free survival (PFS) (7.4 months *vs* 3.7 months; HR 0.66; p<0.0001) and objective response rate (ORR) (40.6% *vs* 12.4%; OR 5.01; p<0.0001) compared with sorafenib (38).

### Second-Line Setting

#### Regorafenib

Regorafenib primarily targets VEGFR1-3, PDGFR-β, FGFR1, Tie-2, c-Kit, RET, B-RAF (40). The FDA approved regorafenib as the second-line setting for advanced HCC in 2017 based on the results of an international, multicenter, randomized, double-blind, placebo-controlled, phase III RESORCE trial (41). The trial aimed to assess the effectiveness and safety of regorafenib in HCC patients who progressed after sorafenib treatment (41). Regorafenib increased OS to 10.6 months from 7.8 months in the placebo arm (HR 0.63; p<0.0001) (41). Regorafenib is the first systemic therapy to show survival benefit in HCC patients who progressed on sorafenib.

#### Cabozantinib

Cabozantinib has dual blocking effects on VEGFR2 and c-Met, which exerts anti-tumor potential by reducing angiogenesis and suppressing cell proliferation, migration and invasion (42). The drug also has targeted inhibition of RET, c-Kit, AXL, FLT3 (43). The randomized phase III clinical trial CELESTIAL enrolled 707 patients with advanced and progressed HCC who had been previously treated with sorafenib (44). Patients in cabozantinib arm showed significantly improvement of survival compared with the placebo arm (median OS: 10.2 months *vs* 8.0 months; HR 0.76; p=0.005. median PFS: 5.2 months *vs* 1.9 months; HR 0.44; p<0.001) (44). Moreover, the ORR in cabozantinib arm was 4%, higher than less than 1% in placebo arm (44). Given the survival benefits brought by cabozantinib, this drug was FDA approved as second-line setting for HCC in 2019.

#### Ramucirumab

Ramucirumab is a fully human IgG1 monoclonal antibody targeting VEGFR2 (45). Unlike small molecule VEGFR TKIs, ramucirumab binds to specific epitope of the extracellular domain of VEGFR2, blocking the binding of the therapeutic target to its ligand VEGF (46). A phase II study showed that ramucirumab 8 mg/kg infused intravenously every 2 weeks had anticancer activity in advanced HCC patients (47). In 2019, the FDA approved ramucirumab as monotherapy for HCC patients having alpha fetoprotein (AFP) ≥400 ng/ml and previously treated with sorafenib. The approval is based on the phase III REACH-2 clinical trial. This is the first positive phase III trial conducted in biomarker-selected HCC patients (48). Both the median OS (8.5 months *vs* 7.3 months; HR 0.710; p=0.0199) and PFS (2.8 months *vs* 1.6 months; HR 0.452; p<0.0001) were longer in ramucirumab arm than that in placebo arm (48). However, there was no statistical difference in ORR between ramucirumab arm (5%) and placebo arm (1%) (p=0.1697) (48). Ramucirumab had a manageable safety and acceptable tolerability. The incidences of serious adverse events were 35% in ramucirumab arm and 29% in placebo arm (48).

## Advances of Other Multi-Targeted Therapeutic Agents for HCC

### Sunitinib

Sunitinib (SU011248) is an oral multi-kinase inhibitor that targets VEGFRs, PDGFRs, c-Kit, FLT3, RET and colony-stimulating factor 1 (CSF-1) (49). The multicenter phase II SAKK 77/06 trial evaluated the antitumor activity of sunitinib in advanced HCC patients (50). Patients were administrated 37.5 mg sunitinib daily until disease progression or intolerable toxicity occurred (50). The stable disease rate was 40% (50). However, another open multicenter phase II study conducted in Europe and Asia reported a low overall ORR (2.7%) in advanced unresectable HCC patients treated with sunitinib, which did not meet the primary endpoint (expected ORR was 15%) (51). In addition, 50 mg/day sunitinib showed severe toxicity (51). Hence, phase III study of sunitinib in HCC was halted due to its toxicity.

### Brivanib

Brivanib is a selective dual inhibitor targeting VEGFR and FGFR. Preclinical study had shown that brivanib significantly inhibited the growth of multiple HCC xenografts (52). Several clinical trials were conducted to evaluate the efficacy of brivanib in advanced HCC patients. In phase II studies, brivanib showed promising antitumor activity as first- or second-line therapy (53, 54). However, brivanib did not significantly improve OS of HCC patients as second-line therapy in phase III study, and another phase III study also did not meet the primary endpoint of OS noninferiority for brivanib *versus* sorafenib (55, 56).

### Vandetanib

Vandetanib is an oral TKI targeting VEGFR, EGFR and RET. In a phase II, randomized, double-blind, placebo-controlled study, vandetanib showed a trend of improvement in PFS and OS for advanced HCC, but there was no statistically significant difference compared to the placebo arm. Also, the two arms had no difference in tumor stabilization rate (57). However, the combination of vandetanib with radiotherapy significantly enhanced radiation killing (58).

### Linifanib

Linifanib (ABT-869) is an ATP-competitive TKI targeting all VEGFRs and PDGFR families (59). In a phase II single-arm clinical trial, linifanib showed clinical activity in advanced HCC patients who had received ≤1 systemic therapy (60). An open-label phase III clinical trial evaluated the efficacy and safety of linifanib *versus* sorafenib in advanced HCC patients who were not systemically treated (61). Although the linifanib arm had longer TTP, PFS and higher response rate, the study did not meet the primary endpoint, with no significant difference in OS between the linifanib and sorafenib arms (61). Moreover, patients in the linifanib arm experienced more frequent grade ≥3 adverse events (61).

### Nintedanib

Nintedanib (BIBF 1120) is an oral triple angiokinase inhibitor targeting VEGFR1-3, FGFR, PDGFR (62). BIBF 1120 (50 or 100 mg/kg/d) showed anti-tumor and anti-angiogenic activity in HepG2 xenograft model (62). In a randomized, multicenter, open-label study of Asian patients with advanced HCC, the phase I portion, patients were divided into two groups based on baseline alanine aminotransferase/aspartate aminotransferase (ALT/AST) and Child-Pugh score (group I: ALT and AST ≤ 2 times the upper limit of normal (ULN) and Child-Pugh score 5-6; group II: ALT or AST>2 to ≤5 times the ULN or Child-Pugh score 7), and the maximum tolerated dose (MTD) of 200 mg was determined for both groups (63). The phase II portion, group I patients were randomly assigned in a 2:1 ratio to nintedanib 200 mg twice daily or sorafenib 400 mg twice daily continuously for 28 days (63). The both arms showed similar results in primary endpoint TTP (2.8 months *vs* 3.7 months) and the secondary endpoint OS (10.2 months *vs* 10.7 months) (63).

### Dovitinib

Dovitinib is a multi-kinase inhibitor targeting VEGFR, PDGFR and FGFR. In addition to its anti-angiogenic effects, dovitinib induces dephosphorylation of retinoblastoma protein, upregulates p-histone H2A-X and p27, and downregulates p-CDK-2 and cyclin B1, thereby reducing cell proliferation and inducing tumor cell apoptosis (64). In addition, dovitinib induces apoptosis of sorafenib-resistant cell lines by inhibiting signal transducer and activator of transcription 3 (STAT3) (65). Unfortunately, a randomized, open-label, phase II study of Asia-Pacific patients reported that dovitinib did not show superior activity to sorafenib in first-line treatment of advanced HCC (66).

### Donafenib

Donafenib is a novel TKI and similar to sorafenib. In a phase Ib clinical trial, a lower dosage of donafenib showed significant anti-cancer effects (TTP was 120 days) and good safety profile in Chinese patients with advanced HCC (67). The ZGDH3 study is the first completed phase II/III clinical trial in China to evaluate the efficacy of donafenib for the first-line treatment of advanced HCC. At the 56th Annual Meeting of the American Society of Clinical Oncology (ASCO 2020), the investigators presented the latest ZGDH3 findings to the world through an oral presentation. The study results showed that the primary endpoint of OS was longer in donafinib arm than sorafenib arm (12.1 months *vs* 10.3 months). The donafenib arm showed a trend toward better overall safety, demonstrating the potential of donafinib in targeted therapy for HCC.

## Potential Therapeutic Targets and Highly Selective Drugs for HCC

### EGF/EGFR

EGFR is a PTK that binds to the ligands EGF and TGF-α to induce receptor dimerization and autophosphorylation, which trigger the downstream MAPK, PI3K, and PLCγ signaling pathways that mediate cell proliferation, survival, adhesion, migration, and differentiation (68–71). EGFR is overexpressed in human HCC cells (72). Some oncogenic mutations such as the L834R mutation lead to spontaneous EGFR dimerization (73). Erlotinib is an oral TKI that specifically blocks tyrosine kinase activity and autophosphorylation of EGFR (74). DCR of 59% was observed in a phase II study of erlotinib for advanced HCC patients who had previously allowed only one systemic or local treatment (74). Bevacizumab plus erlotinib had also shown promising biological activity in the treatment of advanced HCC. In a phase II, single-arm, single-institution, investigator-initiated study, 62.5% of patients were alive and progression free at 16 weeks after the treatment of bevacizumab plus erlotinib (75). The median PFS was 39 weeks, and the median OS was 68 weeks (75).

### FGF19/FGFR4

FGF19 is an important driver of HCC development. It binds to FGFR4 with high affinity (76, 77). Klotho-beta is a co-receptor for FGFR4, which is involved in the activation of FGF19/FGFR4 (78). The FGF19/FGFR4 pathway activates GSK3β/β-catenin, PI3K/AKT, PLCγ/DAG/PKC, RAS/RAF/MAPK signaling cascades and promotes the survival, proliferation, and metastasis of HCC (77). A phase I study evaluated the antitumor activity of fisogatinib (BLU-554), a small molecule highly selective inhibitor targeting FGFR4 (79). The ORR in patients with FGF19-positive tumors was 17%. The median duration of response (DOR) was 5.3 months, and the median PFS was 3.3 months. However, in patients with FGF19-negative tumors, the ORR was 0%, and the median PFS was 2.3 months (79).

### Insulin-Like Growth Factor-1 (IGF-1)/IGF-1 Receptor (IGF-1R)

The binding of ligand IGF-1 to IGF-1R stimulates the activation and phosphorylation of tyrosine kinase, which activates downstream MAPK, AKT and STAT pathways and promotes cell proliferation, migration, stemness and survival (80). Activation of the IGF axis was observed in breast cancer, sarcoma, and non-small cell lung cancer (81). In early HCCs, IGF activity correlated with mTOR signaling and HCC cells proliferation (82). Currently, at least 4 fully human IgG1 monoclonal antibodies targeting IGF-1R have been developed, including cixutumumab (83). The drug blocks phosphorylation of tyrosine residues, mediates receptor internalization and degradation, and produces antibody-dependent complement-mediated cytotoxicity (ADCC) and complement-dependent cytotoxicity (CDC) effects (84). Preclinical study had shown that IGF-1R blockade inhibited the growth of HCC, but no clinically meaningful activity was observed in the phase II study (84, 85). Besides, the combination of cixutumumab and sorafenib also did not exhibit superior clinical efficacy in unselected patients with HCC (86). The IGF-1R is reciprocally activated by NPM-ALK, suggesting that dual inhibition of IGF-1R and ALK could enhance the therapeutic effect of IGF-1R inhibitor (87). Lee reported that cixutumumab treatment activated STAT3 to induce IGF secretion, which recruited macrophages and fibroblasts and created an angiogenic and metastatic environment (88). Therefore, ongoing research elucidating mechanisms of resistance and uncovering responsive biomarkers are required for the success of IGF-1R targeted therapy.

### c-Met

c-Met is an RTK, and its known ligand is HGF (89). HGF induces dimerization and activation of overexpressed c-Met, which stimulates multiple downstream signaling pathways such as MAPK, PI3K, STAT and NF kappa-B (90). In preclinical models of HCC, the HGF/c-Met inhibitor MSC2156119J inhibited tumor growth and induced complete regression (91). Tivantinib (ARQ 197), an orally administered selective c-Met inhibitor, showed antitumor activity in phase I and phase II studies (92, 93). However, in phase III studies, for MET-high advanced HCC patients who previously treated with sorafenib, no significantly improved PFS and OS were observed in tivantinib arm compared to the placebo arm (94, 95). More randomized trials are necessary to determine whether tivantinib is a potential treatment for certain subgroups of patients. Tepotinib, another highly selective c-Met inhibitor, met the primary endpoint in treating sorafenib-pretreated patients with advanced HCC, with a 12-week PFS of 63.3% (96). The HGF/c-Met and VEGF/VEGFR pathways had synergistic effects in neovascularization through enhancing intracellular signaling and modulation of signaling molecules (97). A clinical study reported that advanced HCC patients treated with the anti-VEGFR2 mAb ramucirumab plus the anti-MET mAb emibetuzumab showed an 6.7% overall response rate, 60% DCR and 5.42 months PFS, which further supporting the results of preclinical study (98). In addition, other c-Met inhibitors such as foretinib and capmatinib also showed promising antitumor activity in advanced HCC (99, 100).

### Angiopoetin/Tie-2

Ang-1 and Ang-2 are angiopoietins, which activate Tie-2 receptor and promote neovascularization (101). Trebananib is a peptide inhibitor that blocks the interaction of Ang-1 and Ang-2 with the Tie-2 receptor and reduces tumor angiogenesis (102). The efficacy of trebananib in combination with sorafenib for advanced HCC was evaluated in a phase II study (103). The primary endpoint of the study was planned to be a 4-month PFS of ≥78%. It is disappointing that the study was not met the primary endpoint (103).

### Transforming Growth Factor-β (TGF-β)/TGF-β Receptor (TGF-βR)

TGF-β is a secreted factor that leads to decreased cell adhesion, loss of polarity and tight junctions by inducing epithelial mesenchymal transition (EMT) (104). TGF-β binds to TGF-βR and upregulates the expression of pro-angiogenic factors such as VEGF (104). TGF-β/Smad signaling promotes immune escape by impairing the function of cytotoxic T cells, DC cells and NK cells (104–106). These mechanisms contribute to HCC tumor progression. Galunisertib (LY2157299) is a small molecule inhibitor that selectively targets TGF-βR. This drug demonstrated antitumor activity for second-line treatment of HCC in a phase II study (107). TGF-β/TGFβR signaling has been reported to confer resistance to sorafenib (108). In preclinical study, galunisertib enhanced sorafenib-induced apoptosis (108).

### mTOR

mTOR is a dual-specificity kinase that catalyzes phosphorylation on serine/threonine and tyrosine residues of its substrates (109). mTORC1 and mTORC2 are two major complexes that mediate the regulation of multiple targets by mTOR (109). mTORC1 promotes anabolism of proteins and nucleotides by upregulating the expression of metabolic genes and inhibiting catabolic processes such as autophagy (110). mTORC2 phosphorylates and activates AKT (protein kinase B), PKC (protein kinase C) and SGK (serum/glucocorticoid regulated kinase) of the AGC protein kinase family, which promotes the survival and proliferation of HCC cells (111, 112). In addition, activated AKT phosphorylates and activates mTORC1, resulting in a positive feedback pathway loop that regulates HCC cell growth (110). Preclinical studies showed that mTOR inhibitors significantly inhibit growth and induce apoptosis of HCC cell lines (113–115). Everolimus given daily at 7.5 mg showed clinical activity in advanced HCC patients in a randomized phase I/II study (116). However, in a global multicenter randomized phase III clinical study, everolimus did not improve OS of these patients (117). Treatment of HCC patients undergoing liver transplantation with mTOR-inhibitor temsirolimus for ≥3 months improved survival outcomes, and the greatest benefit was observed in the subgroup with AFP ≥10 ng/ml (118). A phase II trial of bevacizumab plus temsirolimus for the first-line treatment of HCC reported positive results with ORR of 19% and median OS of 14 months (119). However, everolimus plus sorafenib did not demonstrate better survival benefits compared to sorafenib alone in another phase II trial (120). Combination therapy of MEK inhibitors and mTOR inhibitors exhibited enhanced antitumor effects *in vivo* and *in vitro* models of HCC (121).

### Hippo-Yes-Associated Protein (YAP)

The Hippo-YAP pathway plays a prominent role in inhibiting tumor growth, especially in HCC (122). The core component of the Hippo signaling pathway, adaptor protein salvador homolog 1 (SAV1 or WW45), couples mammalian sterile 20-like kinase 1/2 (MST1/2)-mediated kinases large tumor suppressor homolog 1/2 (LATS1/2) phosphorylation (122). This cascade leads to downstream YAP phosphorylation and retention in the cytoplasm, followed by ubiquitination and degradation (122). When Hippo-YAP signaling is attenuated, YAP and transcriptional coactivator translocate to the nucleus and initiate transcription of pro-proliferative and apoptosis-suppressing genes (122). Hypoxia induces nuclear translocation and accumulation of YAP (123). CT-707 is a YAP signaling inhibitor that increases YAP phosphorylation and reduces nuclear accumulation. Both *in vivo* and *in vitro* HCC models have demonstrated potent anti-tumor activity of CT-707 (124).

### RAS/RAF/MEK/ERK

Evidences suggest that the RAS/RAF/MEK/ERK pathway is hyperactive in HCC (125, 126). Activated RAS induces phosphorylation of RAF kinase, which subsequently leads to the phosphorylation of downstream signaling factors MEK and ERK. Phosphorylated ERK dimerizes and translocates to the nucleus to participate in cell proliferation and differentiation (127). Therefore, aberrant activation of the RAS/RAF/MEK/ERK pathway may be critical for the formation and maintenance of HCC. Selumetinib is a small molecule, non-ATP competitive inhibitor that selectively targets MEK1, 2 (128). Disappointingly, in a phase II study of selumetinib for the first-line treatment of advanced HCC patients, no radiographic response was observed and the TTP was short, indicating low monotherapy activity (127). The combination of sorafenib and selumetinib for advanced HCC showed encouraging antitumor activity superior to sorafenib alone in a phase Ib study, suggesting that this combination may have a synergistic effect (129). Several clinical studies had reported that HCC patients treated with the MEK1/2 inhibitor refametinib plus sorafenib had a better clinical response relative to refametinib alone, especially those with RAS mutations (130, 131).

### STAT3

Many cancer cells harbor constitutive activation of STAT3 (132). Phosphorylated STAT3 was detected in 60% of HCC specimens (133). Several cytokines and growth factors such as IL-6, EGF, HGF are involved in the induction of STAT3 activation (134, 135). In addition, phosphorylation of tyrosine residue is critical for STAT3 dimerization, which mediates nuclear entry and DNA binding, inducing target gene transcription (132). Besides, activation of STAT3/SNAIL signaling promotes EMT, contributing to the progression of HCC (136). STAT3 inhibitor OPB-111077 showed limited preliminary efficacy in preclinical HCC models and phase I clinical trial for second-line treatment of advanced HCC (137, 138).

### Endosialin (TEM-1, CD248)

An experiment validated the differential expression of endosialin on tumor-associated myofibroblasts and tumor vessel-associated mural cells, involving in tumor angiogenesis, adhesion to extracellular matrix (ECM) proteins and migration through matrigel (139, 140). Ontuxizumab (MORAb-004-001) is a humanized anti-endosialin IgG1κ monoclonal antibody. The first-in-human study of this drug was conducted in the US as an open-label phase I clinical study for patients with solid tumors who had failed standard chemotherapy. The study observed initial anticancer activity of ontuxizumab (141). A phase I study was subsequently initiated in Japan to confirm the efficacy, safety and tolerability of ontuxizumab in solid tumors. In this study, stable disease rate of 53.3% and tumor shrinkage of 33.3% were observed in HCC patients (142).

### Endoglin (CD105)

Endoglin (CD105) is highly expressed on active endothelial cells (143). Endoglin is involved in angiogenesis, inflammation and cancer-associated fibroblast (CAF) accumulation in the tumor microenvironment (TME) (143). TRC105 is a chimeric IgG1 mAb that competitively blocks the binding of endoglin to its ligand bone morphogenetic protein (BMP) and inhibits tumor angiogenesis (144). TRC105 alone lacked significant clinical activity in the treatment of HCC (145). However, TRC105 in combination with sorafenib showed encouraging activity in first-line treatment of HCC (partial response rate was 25%) (146).

### Cyclin-Dependent Kinase 4/6 (CDK4/6)

CDK4/6 promotes the cell cycle progression (147, 148). CDK4/6 amplification has been found in multiple malignant tumors (149–151). Palbociclib (PD-0332991) is a selective CDK4/6 inhibitor that induces reversible cell cycle arrest in human HCC lines and is efficacious in multiple preclinical models of HCC (152). *In vivo* model, palbociclib in combination with sorafenib was more efficacious than sorafenib alone (152). Another CDK4/6 inhibitor, ribociclib, showed similar antitumor activity in preclinical study (153).

### Histone Deacetylases (HDAC)

HDAC reversibly regulates acetylation of histones and non-histones. Dysregulation and mutation of HDAC lead to abnormal cell proliferation, EMT and tumor angiogenesis (154). Resminostat is a HDAC inhibitor. In the SHELTER study, the combination of resminostat and sorafenib prolonged median TTP and OS compared with resminostat alone (155). However, in comparison of this combination with sorafenib monotherapy for East Asia advanced HCC patients, no significant efficacy advantage was observed in the combination arm (156).

## Combination Therapy of Targeted Therapy and ICI

ICIs is a novel therapeutic approach that differs from conventional treatment mechanisms (157). It restores the viability of tumor-specific T cells and utilizes the host immune system to kill tumors (158, 159). Among many ICIs identified, anti-PD1/PD-L1 and anti-CTLA-4 are currently approved for clinical application, and combination treatment of anti-PD1 and anti-CTLA-4 could have synergistic effect in some kinds of cancer (160–163). PD-L1 expression and tumor mutational burden are widely used molecular marker to guide ICI therapy, but the predictive value is not consistent among different cancers (164, 165). The combination of targeted therapy with ICI shown more potent efficacy (**Table 2**) (32, 166).

**Table 2:** Current clinical trials investigating the combination therapy of targeted agents and ICIs for HCC.

Study design	ClinicalTrials.gov Identifier	Phase	Line	Primary end point	Study status
SHR-1210 + Apatinib	NCT04014101	II	First	ORR	Recruiting
SHR-1210 + Apatinib	NCT03463876	II	Second	ORR	Active, not recruiting
AK104 + Lenvatinib	NCT04444167	Ib/II	First	ORR	Recruiting
Nivolumab + Bevacizumab *vs*. Nivolumab *vs*. Bevacizumab	NCT04393220	II	First	PFS/OS	Recruiting
Pembrolizumab + Regorafenib	NCT04696055	II	Second	ORR	Recruiting
Nivolumab + Galunisertib	NCT02423343	I/II	Second	MTD	Completed
Toripalimab + ATG-008	NCT04337463	I	Second	MTD/RP2D/ORR	Recruiting
HLX10 + HLX04	NCT03973112	II	Second	ORR	Recruiting
HX008+Bevacizumab *vs*. HX008 + Lenvatinib	NCT04741165	II	First	ORR	Recruiting
Sintilimab + Lenvatinib	NCT04042805	II	First	ORR	Recruiting
Toripalimab + Lenvatinib	NCT04368078	II	Second	ORR	Recruiting
Toripalimab + Bevacizumab	NCT04605796	II	First	ORR/Safety	Recruiting
Camrelizumab + Lenvatinib	NCT04443309	I/II	First	ORR	Recruiting
Camrelizumab + Apatinib	NCT04701060	II	First	ORR	Recruiting
Tislelizumab + regorafenib *vs*. regorafenib	NCT04183088	II	First	ORR/PFS	Recruiting
MK-1308A + Lenvatinib	NCT04740307	II	First	ORR	Recruiting
Pembrolizumab **+** Lenvatinib *vs*. Lenvatinib + placeco	NCT03713593	III	First	PFS/OS	Active, not recruiting
Nivolumab + Lenvatinib	NCT03841201	II	First	ORR/Safety	Recruiting
PDR001 + Sorafenib	NCT02988440	I	First	AE/DLT	Completed
Atezolizumab + Bevacizumab	NCT04102098	III	First	RFS	Recruiting
Avelumab + Axitinib	NCT03289533	I	First	AE	Completed
Atezolizumab + Cabozantinib *vs*. sorafenib	NCT03755791	III	First	PFS/OS	Recruiting
Durvalumab + Tivozanib	NCT03970616	I/II	First	AE	Recruiting
Durvalumab + Bevacizumab *vs*. Durvalumab	NCT03847428	III	First	RFS	Recruiting

ICIs, immune checkpoint inhibitors; HCC, hepatocellular carcinoma; ORR, objective response rate; PFS, progression-free survival; OS, overall survival; MTD, maximum tolerated dose; RP2D, recommended phase II dose; AE, adverse event; DLT, dose limited toxicity; RFS, recurrence-free survival.

Encouraging results from the CheckMate-040 (167) and KEYNOTE-224 (168) studies led to accelerated FDA approval of nivolumab and pembrolizumab as second-line therapy for advanced HCC. Further, combination of targeted therapy with immunotherapy becomes mainstream, especially anti-angiogenesis therapy and ICI (169). In multiple mice models, combinations of ICI with anti-angiogenesis agents significantly increase the active anti-tumor immune cell and reduce the immune inhibitory components in comparison with ICI alone. At present, it is well accepted that combination therapy of ICI and anti-angiogenesis could achieve superior efficacy to monotherapy in several types of solid cancer (170). Atezolizumab is a high-affinity human monoclonal IgG1 antibody that specifically targets PD-L1 and blocks its interaction with PD-1 and B7.1, recovering pre-existing anti-tumor immunity (164, 171). Bevacizumab is an anti-VEGF monoclonal antibody (172). In a phase II trial, 13% ORR was observed in bevacizumab-treated patients with unresectable, nonmetastatic HCC (172). Results from a multiarm phase Ib GO30140 study suggested atezolizumab plus bevacizumab had a more significant PFS benefit than atezolizumab alone (173). On May 29, 2020, the FDA approved atezolizumab plus bevacizumab as the first-line setting for patients with unresectable or metastatic HCC. Approval was granted following the results of phase III IMbrave150 trial (32). This trial assessed the efficacy of atezolizumab plus bevacizumab *versus* sorafenib and demonstrated that atezolizumab plus bevacizumab arm had higher 12-month OS (67.2% *vs* 54.6%) and longer PFS (6.8 months *vs* 4.3 months; HR 0.59; *p*<0.001) than sorafenib arm (32). The incidences of grade 3/4 adverse events were 56.5% with atezolizumab-bevacizumab and 55.1% with sorafenib (32). Approval of atezolizumab plus bevacizumab is likely to change the paradigm of the treatment of HCC. In a phase Ib study, lenvatinib plus the anti-PD-1 mAb pembrolizumab had promising anticancer activity in advanced HCC. The ORR and DOR were 46.0% and 8.6 months, respectively. The median PFS and OS were 8.6 months and 22 months, respectively (174). The combination of ramucirumab and the anti-PD-L1 mAb durvalumab also showed promising results in a phase Ia/b open-label study of advanced HCC. The ORR was 11%. The median PFS and OS were 4.4 and 10.7 months, respectively (175). SHR-1210 (anti-PD-1 antibody) 200 mg every 2 weeks plus apatinib 250 mg daily exhibited encouraging clinical activity in advanced HCC in an open, dose-escalation and extension study (176). The ORR was 30.8% and partial response was achieved in 8 of 16 evaluable HCC patients (176). Clinical trials of other targeted drugs in combination with ICIs are also underway. In ASCO 2021, the preliminary results of some ongoing clinical trials showed that combination therapies of ICIs with anlotinib had superior efficacies to monotherapies (177, 178). In addition, studies demonstrated that PARP inhibitors could also enhance the efficacy of ICIs by promoting antigen presentation and modifying immune microenvironment, leading to the enhanced tumor-killing activities of T cell (179).

## Conclusion and Perspective

Advanced HCC is a major challenge in cancer treatment. Sorafenib is the first FDA-approved TKI for the first-line treatment of advanced HCC, bringing a breakthrough to the treatment challenge. Based on the promising results in clinical studies, other molecularly targeted drugs such as lenvatinib, regorafenib, cabozantinib, ramucirumab also have been approved by FDA for first- or second-line treatment of advanced HCC. However, the efficacy is far from being satisfied. Therefore, new targets are extensively explored. In addition to interfering with the interaction between PTK and ligand, blocking the downstream signaling pathway of PTK cascade also exhibits effective inhibition of HCC progression, such as mTOR inhibitors, MEK inhibitors and STAT3 inhibitors. Besides, targeted inhibitors acting on cell cycle progression also show antitumor potential in preclinical studies of HCC. Following the research advance, potential target for HCC continues to be uncovered. For example, a recent study demonstrated that p38 MAPK gamma induced mouse hepatocyte proliferation after partial hepatectomy by promoting the phosphorylation of retinoblastoma protein as CDK-like kinase. Moreover, p38γ was required for the chemically induced formation of liver tumors (180). Sterol o-acyltransferase1 (SOAT1) and carnitine palmitoyltransferase 1A (CPT1A) were found to regulate fatty acid metabolism, and simultaneously targeting SOAT1 and CPT1A demonstrated synergistic anticancer efficacy in HCC *in vitro* and *in vivo* models (181). Liu et al. applied multi-omics technology to characterize tumor microenvironment and defined HCC into three immune subtypes. Their study suggested that MMP-9 reflected immune features and might be a valuable predictor of immunotherapeutic response in HCC (182).

Despite impressive progress in targeted therapy for advanced HCC, several challenges remain. One is drug-related adverse events, which lead to dose reduction, interruption or discontinuation. Besides, drug resistance remains a major cause of the failure of targeted therapy. The underlying mechanisms may be tumor heterogeneity and clonal evolution. In addition, there is a lack of reliable biomarkers to identify the HCC patients most likely to benefit from targeted therapy. Some circulating markers, such as AFP, IL-6 and TNF-α, correlate with the treatment outcomes of HCC (183–185), but large prospective studies are required to validate the preliminary findings. How to overcome these challenges and explore low-toxic and efficient treatment strategies are the direction of effort.

Single drug activity is insufficient and a rational combination of different drugs is needed to obtain maximum benefit. The combination of targeted therapy plus ICI has attracted attention, with positive results in several clinical trials. In the future, the integration of multidisciplinary treatment approaches for advanced HCC and the development of personalized treatment plans based on the disease status of HCC will contribute to the progress of precision medicine.

## Author Contributions

MN drafted the manuscript and prepared the figure and tables. MY and NL helped in revising it critically for important intellectual content. KJW and KMW designed this review and revised the manuscript. All authors contributed to the article and approved the submitted version.

## Funding

This work was supported by the National Natural Science Foundation of China (No.81874120, 82073370).

## Conflict of Interest

The authors declare that the research was conducted in the absence of any commercial or financial relationships that could be construed as a potential conflict of interest.

## Publisher’s Note

All claims expressed in this article are solely those of the authors and do not necessarily represent those of their affiliated organizations, or those of the publisher, the editors and the reviewers. Any product that may be evaluated in this article, or claim that may be made by its manufacturer, is not guaranteed or endorsed by the publisher.
